# Effects of grazing on plant functional groups across spatial scales in *Stipa breviflora* desert steppe

**DOI:** 10.3389/fpls.2025.1643655

**Published:** 2025-09-18

**Authors:** Li Wang, Xiaoyu Du, Juhong Liu, Jun Zhang, Shijie Lv

**Affiliations:** ^1^ College of Science, Inner Mongolia Agricultural University, Hohhot, China; ^2^ College of Grassland Science, Inner Mongolia Agricultural University, Hohhot, China

**Keywords:** desert steppe, spatial scale, heavy grazing, plant functional group, interspecific associations

## Abstract

This study investigated the *Stipa breviflora* desert steppe through multi-scale (50m×50m, 25m×25m, 2.5m×2.5m) and grazing intensity (no grazing vs. heavy grazing) comparative analyses, revealing the response mechanisms of plant functional group diversity, interspecific associations, and stability. Key findings include: (1) Heavy grazing significantly reduced functional group diversity and evenness, while the Margalef richness index increased at the 25m×25m scale due to patchy invasion of grazing-tolerant species. (2) Interspecific associations exhibited scale-dependent patterns: Large-scale (50m×50m) associations were driven by environmental heterogeneity (e.g., resource competition and complementarity), whereas small-scale (2.5m×2.5m) interactions were dominated by direct species interactions (mutualism or exclusion). (3) Grazing-induced structural simplification through “environmental filtering”, heavy grazing reduced functional group quantity, forming simplified symbiotic networks (PC≥0.6) between perennial grasses and annual/biennial plants, while significantly suppressing woody plants and forbs (Perennial forbs, Shrubs and semi-shrubs). (4) Stability analysis demonstrated higher stability of perennial grasses and forbs in ungrazed areas, though the overall system remained unstable. Annual/biennial plants and shrubs/semi-shrubs generally exhibited low disturbance resistance. The study proposes a multi-scale grassland restoration strategy: optimizing resource allocation at large scales while enhancing key species interactions at small scales. These findings provide theoretical foundations for the ecological restoration of degraded desert steppes and adaptive grazing regimes. Future research should integrate climate change and socioeconomic factors to develop more resilient grassland ecosystem management frameworks.

## 1 Introduction

The *Stipa breviflora* desert steppe in Inner Mongolia is a vital grassland ecosystem, crucial for livestock production and ecological security (Wang Y. et al., 2023). Characterized by arid to semi-arid features, it is dominated by Stipa breviflora, with species like *Cleistogenes songorica* and *Artemisia frigida*, forming a unique plant functional group. As an ecologically fragile zone, it is highly sensitive to grazing, with overgrazing causing vegetation degradation and biodiversity loss (Du et al., 2024).

In desert steppe ecosystems, a “functional group” refers to a combination of plant species sharing similar ecological functional traits (Zhang et al., 2024). Vegetation in the *Stipa breviflora* desert steppe can be classified into functional groups based on life forms (e.g., Perennial grasses, Perennial forbs, Annual and biennial plants, Shrubs and semi-shrubs) (Wang et al., 2020) or ecological guilds (e.g., xerophytes vs. meso-xerophytes).

Grazing intensity has a significant effect on the importance value of *Stipa breviflora* and other species. It was shown that the competitive advantage of *Stipa breviflora* significantly increased under heavy grazing conditions, and the importance value also increased (Li et al., 2022b; Wang et al., 2025). Grazing simplifies communities through environmental filtering (Ju et al., 2024). Plant communities in ungrazed areas exhibited relatively high stability, primarily attributed to their higher species diversity, more complex community structure, and undisturbed resource allocation and interspecific relationships (Du et al., 2024).

The increase in grazing intensity leads to a significant decline in plant diversity and ecological functions (Wang Z. et al., 2023; Lv et al., 2020; Zhang et al., 2021). The mechanism stems from selective feeding reduces palatable species, lowers litter input, and weakens the soil carbon and nitrogen cycle (Morin et al., 2014; del Río et al., 2017). Grazing inhibits the stability of dominant species and weakens the compensation effect between functional groups, thereby reducing the ecosystem’s resistance (Wang et al., 2020).

It was also found that there is a complex relationship between grazing intensity and the structural stability of plant communities. The structural stability of plant communities was higher under light grazing conditions and significantly lower under heavy grazing conditions (Lv et al., 2024).

The importance of plants and their relationship with grazing intensity showed significant differences at different spatial scales. At smaller spatial scales (e.g., 2.5 m × 2.5 m), the dominance of *Stipa breviflora* was more pronounced, whereas at larger spatial scales (e.g., 50 m × 50 m), the dominance of other species, such as saltbush, was more pronounced (Du et al., 2024). This phenomenon suggests that spatial scale has an important influence on the structure and function of plant communities, especially under different grazing intensities.

However, existing studies have some limitations, most studies focus on a single ecological indicator (such as the density of a certain species) or a single functional group, ignoring the multi-dimensionality and spatial scale dependence of ecosystem responses (Donohue et al., 2013; Kang et al., 2020). The mechanism by which grazing affects functional groups is complex and may be interactively regulated by spatial scale, interspecific interactions, and environmental factors, but the mechanism of such cross-scale interactions remains unclear (Joubert et al., 2017; Fayiah et al., 2019). This makes the differentiated grazing management strategies based on functional group responses and applicable to different spatial scales lack a solid theoretical foundation (Yang et al., 2021; Gerhard et al., 2022).

Therefore, understanding grazing management strategies at different spatial scales is essential to maintain the health of grassland ecosystems. Based on this, this study proposes: Hypothesis 1: Heavy grazing simplifies the composition of functional groups through environmental filtering, but at a moderate scale (25m×25m), diversity “peaks” occur due to plaque dynamics; Hypothesis 2: Large-scale (50m×50m) interspecific associations are dominated by resource competition, while small-scale (2.5m×2.5m) ones are driven by microhabitat interactions. Hypothesis 3: Perennial herbaceous and hybrid grasses have higher stability in herdless areas, but the system as a whole is still in a non-equilibrium state.

To verify this, this study establishes a multi-scale experimental design across the *Stipa breviflora* desert steppe, incorporating contrasting spatial scales (2.5m×2.5m, 25m×25m, 50m×50m) and grazing intensities (ungrazed vs. heavily grazed) to systematically address the following scientific inquiries:

1. How does heavy grazing modify diversity attributes of plant functional groups across spatial scales?
2. Do interspecific association patterns among functional groups exhibit scale dependency under grazing disturbance?
3. How does the stability of distinct functional groups vary with spatial scale and grazing intensity?

The research aims to elucidate the synergistic regulatory mechanisms through which grazing and spatial scales jointly govern plant functional group structure, interaction networks, and stability, thereby providing theoretical foundations for multi-scale restoration and adaptive management of degraded grasslands. Concurrently, stability analysis reveals the disturbance resistance capacities of key functional groups and their contributions to ecosystem resilience. The findings not only advance mechanistic understanding of grassland ecosystem degradation but also provide scientific substantiation for optimizing grazing regimes and formulating differentiated restoration strategies.

## 2 Materials and methods

### 2.1 Study site description

The study area is located in the Siziwang base of the Comprehensive Experimental Demonstration Center of the Inner Mongolia Academy of Agricultural and Animal Husbandry Sciences (41°47′17″N, 111°53′46″E, elevation 1450 m), which belongs to the short-flowered coniferous desert steppe zone, and the climate is a medium-temperate continental type. The average annual precipitation is 280 mm, evapotranspiration is 2300 mm, the average annual temperature is 3.4°C, ≥10°C cumulative temperature is 2200~2500°C, the frost-free period is 90~120 days, and the soil is dominated by light chestnut-calcium soil, which provides a typical environment for exploring the spatial response of the vegetation to the rate of livestock carrying.

### 2.2 Experimental setup

**Figure 1:**
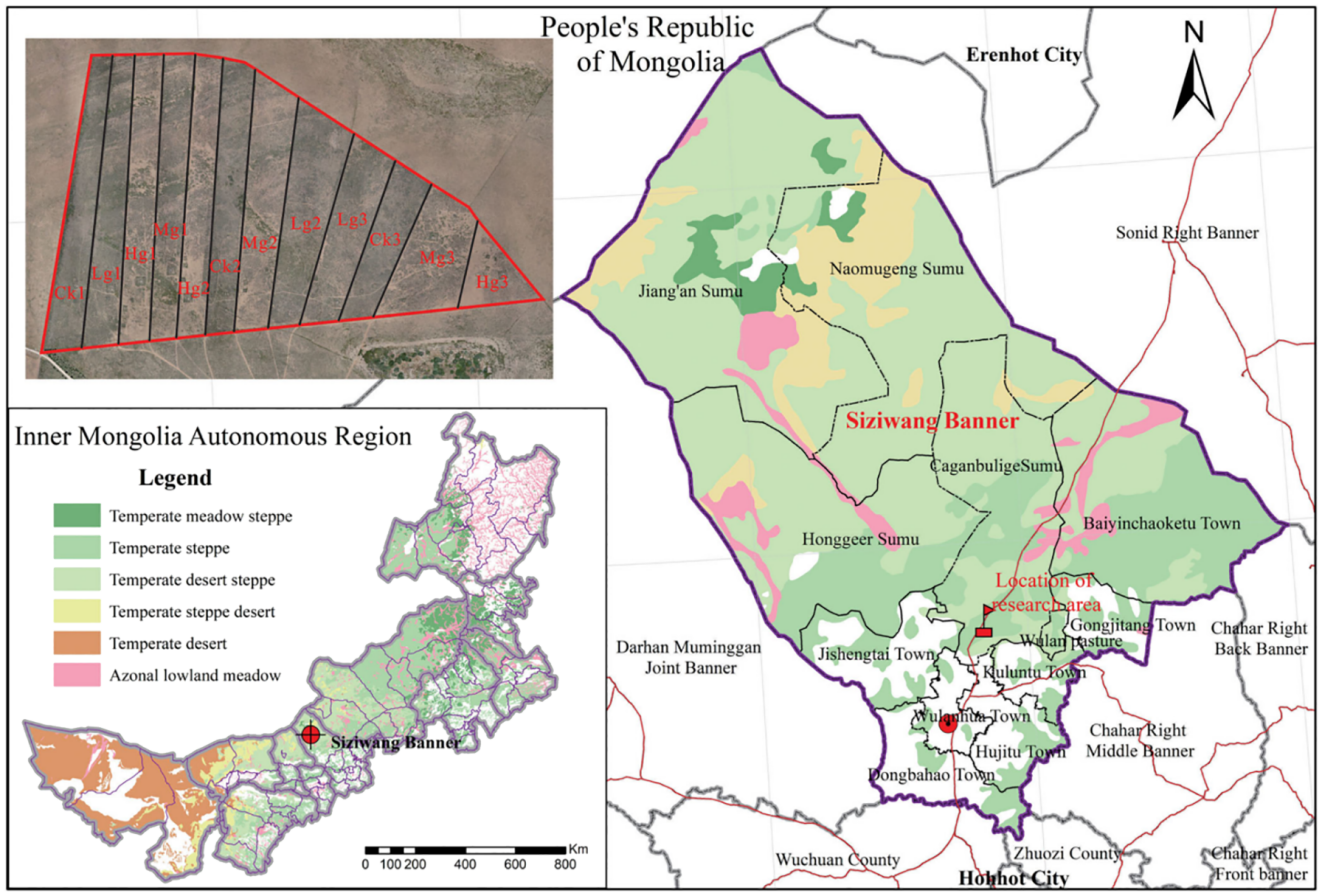
The location of Siziwang Banner in inner Mongolia and experiment plots in Siziwang Banner.

In this study, a grazing control experiment was established in June 2023 in a meadow in northern China, with a total test area of 8.4 ha, and two gradient treatments were set up: a no-grazing control (CK) and heavy grazing (HG) (**Figure 1**) (Zhang et al., 2023). Among them, the HG treatment unit was configured at a loading rate of 0.45 sheep hectare^-1^ month^-1^ and 12 healthy adult sheep were placed in each independent sample plot (about 26.7 ha). CK1 and HG1 were selected as the core observation plots, and the grazing intervention period covered the complete plant growing season (June-November), with 12 hours (06:00-18:00) of continuous grazing per day. All experimental sheep were uniformly bred as two-year-old de-stemmed rams to ensure the consistency of individual physiological status and feeding behavior.

**Figure 2:**
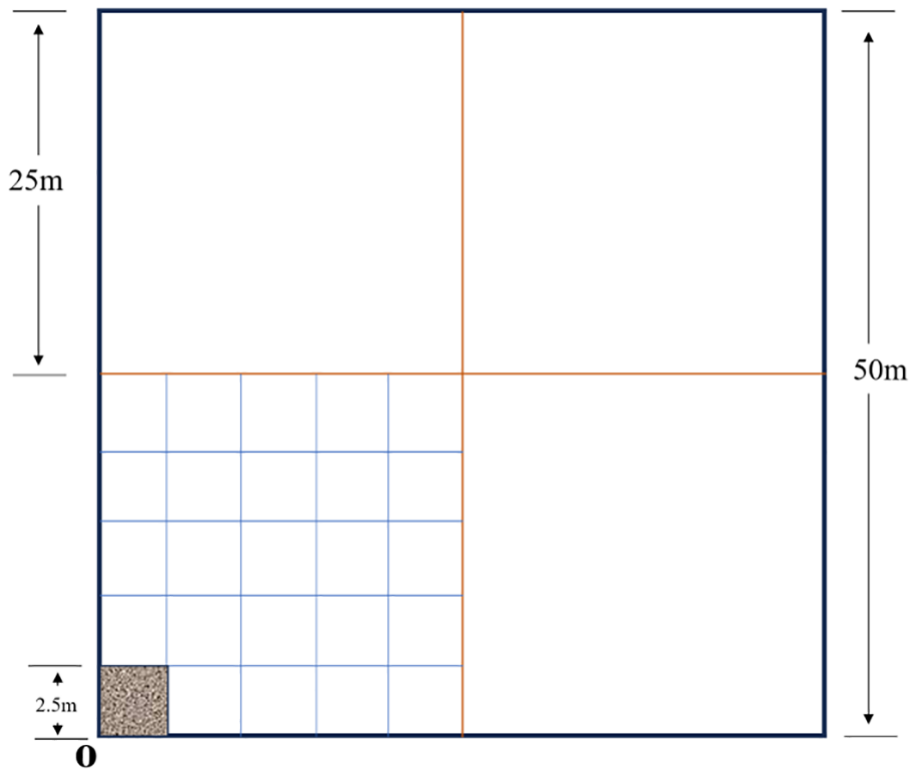
The diagram of the spatial scale plot.

The study analyzed the vegetation distribution pattern through nested multi-scale sampling: a 50 m × 50 m main sample plot (vertex as the coordinate origin) was set up in the loading rate experimental area, and 25 m × 25 m (5 m × 5 m grid) and 2.5 m × 2.5 m sample plots were nested sequentially, the latter matching plant coordinates through a virtual grid (0.25 m to 0.05 m scale), combining field measurements with computer analysis to collect 36 grid vertex data (species, height, biomass). The data integration covered both macro (50 m/25 m) and micro (below 2.5 m) scales to analyze the variation thresholds of interspecific competition intensity with spatial scales, and to reveal the mechanism of the influence of stocking rate on the spatial pattern of vegetation (**Figure 2**).

The plant community of the *Stipa breviflora* desert grassland is divided into four functional groups based on life type and ecological function, and each functional group maintains the stability of the ecosystem through synergistic effects (Wang et al., 2020). The perennial grasses are the established functional groups in this grassland, with *Stipa breviflora* as the dominant species. The functional groups of *Stipa breviflora* desert grassland are categorized as follows (**Table 1**).

**Table 1:** Functional group classification.

Functional groups	Perennial grasses	Perennial forbs	Annual and biennial plants	Shrubs and semi-shrubs
Species	*Stipa breviflora*	*Convolvulus ammannii*	*Teloxys aristata*	*Caragana stenophylla*
	*Cleistogenes songorica*	*Lagochilus ilicifolius*	*Salsola collina*	*Krascheninnikovia ceratoides*
	*Leymus chinensis*	*Allium tenuissimum*	*Euphorbia humifusa*	*Bassia prostrata*
	*Cleistogenes squarrosa*	*Sibbaldianthe bifurca*	*Oxybasis glauca*	*Artemisia frigida*
	*Stipa sareptana* var. *krylovii*	*Asparagus cochinchinensis*	*Neopallasia pectinata*	

### 2.3 Data analysis

#### 2.3.1 Diversity indices calculation

To assess the structure and diversity of plant functional groups, four widely used indices were calculated:

##### 2.3.1.1 Shannon-Wiener diversity index


$$H^{\prime }=-\sum_{i=1}^{S}\left(p_{i}\ln p_{i}\right)$$

##### 2.3.1.2 Simpson dominance index


$$D=1-\sum_{i=1}^{S}p_{i}^{2}$$

##### 2.3.1.3 Margalef richness index


$$R=\frac{S-1}{\ln N}$$

##### 2.3.1.4 Pielou evenness index


$$J^{\prime }=\frac{H^{\prime }}{\ln S}$$

The Shannon-Wiener index $H^{\prime }$ ranges between $0\leq H^{'}\leq \ln S$ , where higher values reflect communities with greater species diversity and more balanced resource allocation. The Simpson dominance index D quantifies the degree of resource monopolization by dominant species within a community. Its value ranges between 0≤D≤1 , where values approaching 1 indicate strong dominance by one or few species. The Margalef richness index R standardizes species richness relative to sampling effort, enabling cross-habitat comparisons. The Pielou evenness index $J^{\prime }$ evaluates the uniformity of species abundance distribution. Its value ranges from $0\leq J^{\prime }\leq 1$ where values approaching 1 indicate near-perfect resource homogenization, while lower values suggest skewed distributions favoring dominant species.

#### 2.3.2 Interspecific association

##### 2.3.2.1 Overall interspecific association

The calculation formulas are as follows:


$$VR=\frac{S_{T}^{2}}{\sigma_{T}^{2}}$$

$$S_{T}^{2}=\frac{1}{N}\sum_{j=1}^{N}{\left(s_{j}-\bar{s}\right)}^{2}$$

$$\sigma_{T}^{2}=\sum_{i=1}^{S}p_{i}\left(1-p_{i}\right)$$

Statistic W=VR×N , if $W\notin \left[\chi_{0.05}^{2}\left(N\right),\chi_{0.95}^{2}\left(N\right)\right]$ , then the overall association is significant (Du et al., 2024).

##### 2.3.2.2 Chi-square test

The Chi-square test evaluates whether the distributions of two species across quadrats are statistically independent, determining the significance of interspecific associations using 2×2 contingency tables. The calculation formulas are as follows:


$$\chi^{2}=\frac{{\left(\left|ad-bc\right|-N/2\right)}^{2}N}{\left(a+b\right)\left(c+d\right)\left(a+c\right)\left(b+d\right)}$$

When $\chi^{2}<3.841$ ( P>0.05 ) represents no significant association When $3.841<\chi^{2}<6.635$ ( 0.01<P<0.05 ) represents significant association. When $\chi^{2}>6.635$ ( P<0.01 ) represents highly significant association (Du et al., 2024).

##### 2.3.2.3 Association coefficient

AC quantifies the direction and strength of species associations -1 to 1, addressing the limitations of Chi-square in sample size dependency. AC→1 means strong positive association. AC→−1 means strong negative association (Du et al., 2024). Three calculation scenarios based on contingency tables


$$Case1\;ad\geq bc,AC=\frac{ad-bc}{\left(a+b\right)\left(b+d\right)}$$

$$Case2\;bc>ad\;and\;d\geq a,\;AC=\frac{ad-bc}{\left(a+b\right)\left(a+c\right)}$$

$$Case3\;bc>ad\;and\;d<a,\;AC=\frac{ad-bc}{\left(b+d\right)\left(d+c\right)}$$

##### 2.3.2.4 Percentage co-occurrence

PC measures the relative frequency of co-occurrence events, ignoring mutual absence d. Higher PC indicates stronger positive associations. The calculation formulas are as follows:


$$PC=\frac{a}{a+b+c}$$

##### 2.3.2.5 Ochiai index

A standardized measure of co-occurrence sensitivity, ranging from 0 to 1. Higher OI indicates stronger associations. The calculation formulas are as follows:


$$OI=\frac{a}{\sqrt{\left(a+b\right)\left(a+c\right)}}$$

##### 2.3.2.6 Dice index

Standardizes co-occurrence rate while ignoring mutual absence d . Higher DI indicates stronger associations. The calculation formulas are as follows:


$$DI=\frac{2a}{2a+b+c}$$

#### 2.3.3 Interspecific correlation

1. Pearson correlation coefficient: Measures linear correlation between species abundances (biomass or density), ranging from -1 to 1.
2. Spearman’s rank correlation coefficient: A non-parametric measure of monotonic relationships between ranked abundance data.

#### 2.3.4 Stability

The stability of functional groups was assessed using the Godron stability index by analyzing species occurrence frequencies. All plant species within the functional groups were ranked according to their occurrence frequencies. Cumulative inverse percentages and cumulative relative frequencies were subsequently calculated. These paired parameters were plotted to construct a graphical model. The intersection coordinates between the model curve and the reference line y=100−x were identified. Stability was quantified based on the proximity of these coordinates to the theoretical equilibrium point (Davison et al., 2020; Tran et al., 2021), with closer distances indicating higher functional group stability (Du et al., 2024).

### 2.4 Spatial structure analysis

To quantitatively verify the sensitivity of the sampling scale and reveal the spatial pattern changes caused by grazing, we adopted geostatistical semi-variogram analysis. Based on the importance values of plant functional groups and their exact coordinates of each grid vertex within splines, the empirical semi-variation function was calculated.

## 3 Results

### 3.1 Functional group classification and diversity in *Stipa breviflora* desert steppe

Significant differences in functional group diversity indices were observed across grazing treatments. At all spatial scales, heavy grazing exhibited a reduced Shannon-Wiener diversity index (H’), Simpson’s dominance index (D), and Pielou’s evenness index (J’) compared to control groups. However, at the 25m×25m scale, heavy grazing showed an elevated Margalef richness index relative to controls.

The effects of spatial scale on functional group diversity were non-significant. At the finer 2.5m×25m scale, species diversity indices were lower than those at broader scales (50m×50m and 25m×25m). Nevertheless, minimal divergence occurred between 50m×50m and 25m×25m scales, indicating relatively weak scale-dependent impacts on diversity within larger spatial extents (**Table 2**).

**Table 2:** Functional group diversity indices at different spatial scales with different grazing levels.

Diversity indices	50m×50m		25m×25m		2.5m×2.5m	
	No grazing	Heavy grazing	No grazing	Heavy grazing	No Grazing	Heavy grazing
Margalef richness index	1.108	1.028	1.108	1.542	1.251	1.116
Shannon-Wiener diversity index	0.599	0.436	0.599	0.587	0.583	0.439
Simpson dominance index	0.747	0.612	0.747	0.735	0.727	0.611
Pielou evenness index	0.432	0.397	0.432	0.423	0.420	0.400

### 3.2 Importance values

At different spatial scales, perennial grasses had the highest importance values when there was no grazing, and under heavy grazing conditions, perennial grasses continued to have higher importance values, but the importance values of Annual and biennial plants increased significantly, and those of perennial forbs and shrubs and semi-scrubs decreased significantly (**Table 3**).

**Table 3:** Importance values of functional groups at different spatial scales and different grazing intensities.

Functional groups	50m×50m		25m×25m		2.5m×2.5m	
Importance values	No grazing	Heavy grazing	No grazing	Heavy grazing	No grazing	Heavy grazing
Perennial grasses	0.56	0.54	0.45	0.57	0.62	0.63
Perennial forbs	0.20	0.04	0.25	0.04	0.17	0.01
Annual and biennial plants	0.06	0.42	0.08	0.39	0.02	0.37
Shrubs and semi-shrubs	0.17	0.00	0.22	0.00	0.18	0.00

### 3.3 Overall interspecific association

The Across spatial scales, the overall interspecific association (variance ratio, VR) in heavily grazed areas consistently exceeded that in ungrazed areas (**Table 4**).

**Table 4:** The overall association of desert grasslands at different spatial scales and different grazing intensities.

Spatial scale	Habitat type	Variance ratio	Statistic W	(Chi0.95, Chi0.05)	Association
50m×50m	No Grazing	0.76	27.36	(23.27, 51.00)	Negative correlation, not significant
	Heavy Grazing	1	36	(23.27, 51.00)	No correlation, not significant
25m×25m	No Grazing	1.32	38.04	(23.27, 51.00)	Positive correlation, not significant
	Heavy Grazing	1.11	39.82	(23.27, 51.00)	Positive correlation, not significant
2.5m×2.5m	No Grazing	0.92	23.09	(14.61, 37.65)	Negative correlation, not significant
	Heavy Grazing	1	25	(14.61, 37.65)	No correlation, not significant

At a scale of 50m×50m, under non-grazing conditions, there was an overall insignificant negative correlation among functional groups (VR< 1), while under heavy grazing, there was no correlation (VR = 1), and it was also insignificant. This indicates that on a larger scale, there is interspecific competition for environmental resources among functional groups in ungrazed areas, and grazing may alleviate this competitive relationship to a certain extent. At the 25m×25m scale, the functional groups under both grazing treatments showed an insignificant positive correlation (VR > 1), and the VR value under heavy grazing was higher than that under non-grazing conditions. At a fine scale of 2.5m×2.5m, ungrazing still showed an insignificant negative correlation (VR< 1); However, heavy grazing triggered a significant positive association (VR > 1, P< 0.05). This result indicates that on a smaller spatial scale, grazing may have promoted facilitating interactions among functional groups or significantly altered the original competitive landscape.

### 3.4 Interspecific association

#### 3.4.1 Chi-square test ( $\chi^{2}$ Test)

**Figure 3:**
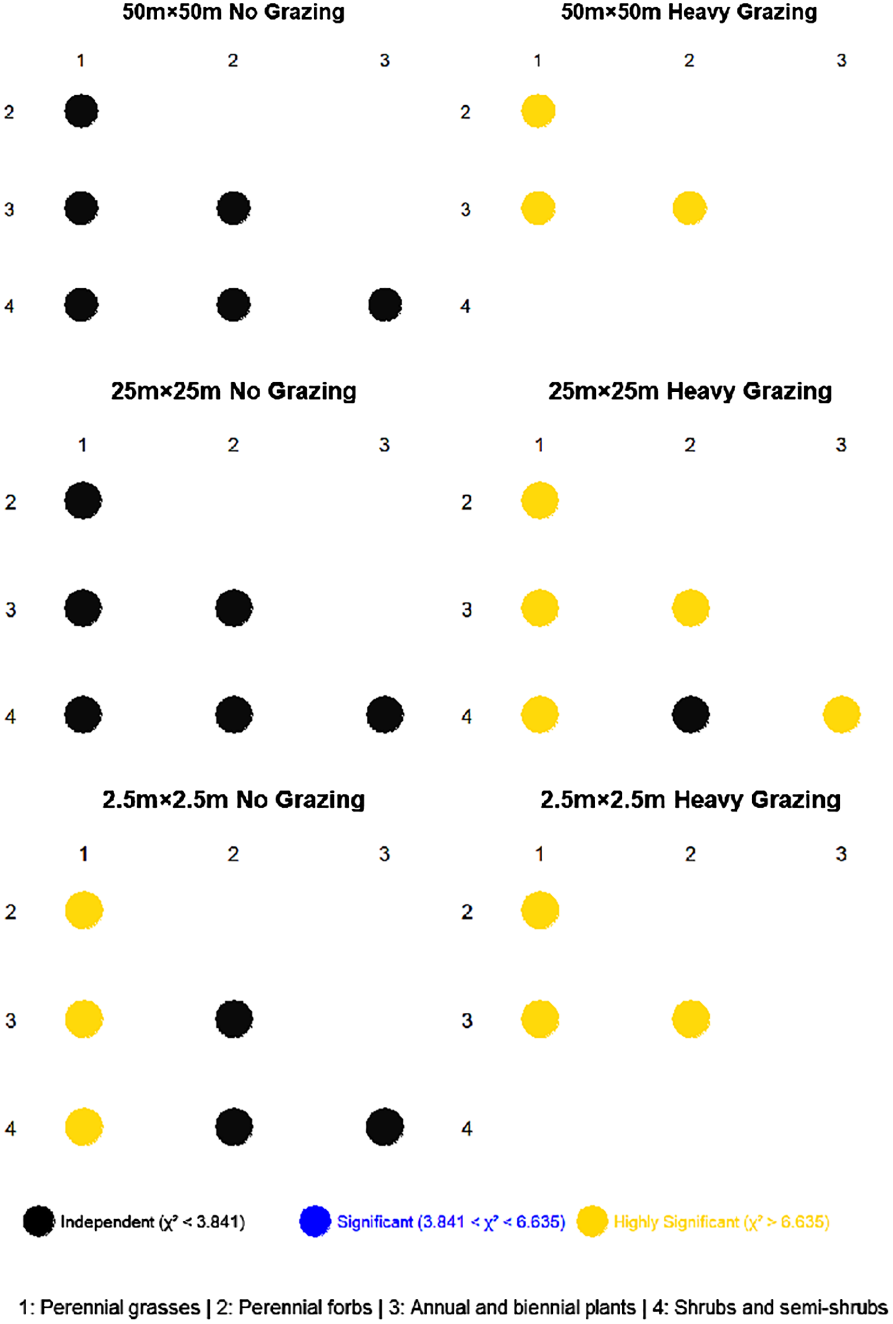
Chi-square (χ²) tests at different scales under different grazing intensities.

At the 50m×50m spatial scale (**Figure 3**), functional groups in the ungrazed area exhibited no significant associations, whereas, in the heavily grazed area, highly significant associations were observed among functional groups. Specifically, annual/biennial plants showed a positive association with perennial grasses, but negative associations with perennial forbs. Additionally, a negative association existed between perennial grasses and perennial forbs.

At the 25m×25m spatial scale (**Figure 3**), no significant associations were detected among functional groups in the ungrazed area. In contrast, under heavy grazing, highly significant associations were identified among most functional groups, except between perennial forbs and shrub/semi-shrub groups, which showed no association. Perennial grasses displayed a positive association with annual/biennial plants, while all other functional group pairs exhibited negative associations.

At the 2.5m×2.5m spatial scale (**Figure 3**), the ungrazed area demonstrated highly significant positive associations between perennial grasses and both perennial forbs and shrub/semi-shrub groups, alongside a highly significant negative association (P< 0.001) between perennial grasses and annual/biennial plants. Associations among other functional groups were non-significant. In the heavily grazed area, all functional groups showed highly significant associations. A positive association was observed between perennial grasses and annual/biennial plants, while negative associations occurred between perennial grasses and perennial forbs, as well as between annual/biennial plants and perennial forbs.

#### 3.4.2 Association coefficient

**Figure 4:**
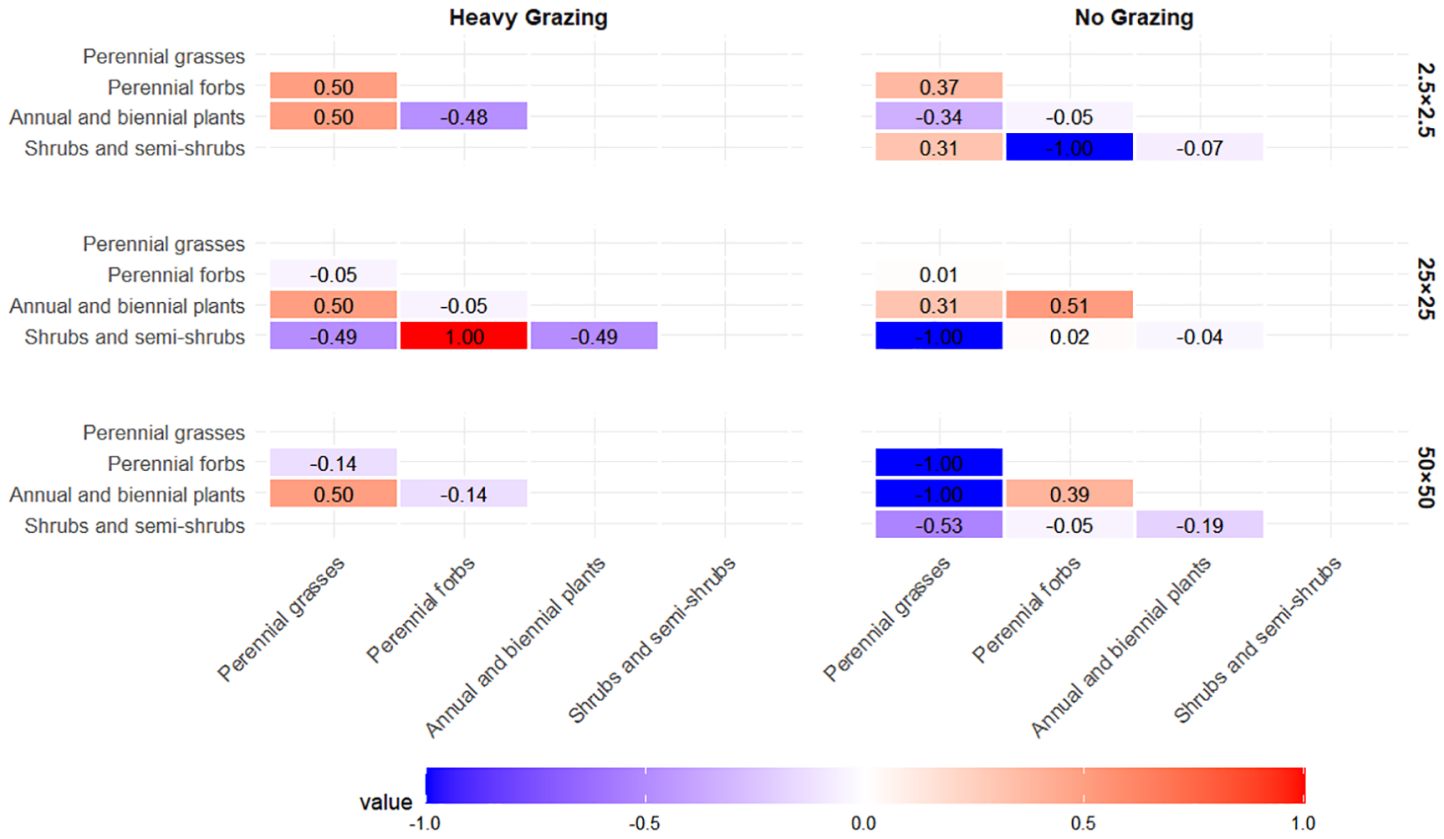
Association coefficient (AC) values at the 2.5m×2.5m scale under different grazing intensities.

At the 50m×50m spatial scale (**Figure 4**), distinct negative associations were observed between perennial grasses and both perennial forbs and shrub/semi-shrub groups in the ungrazed area, while a weak positive association existed between perennial forbs and shrub/semi-shrub groups. Annual/biennial plants exhibited weak negative associations with all other functional groups. In the heavily grazed area, annual/biennial plants displayed a weak positive association with perennial grasses, whereas weak negative correlations were observed between perennial forbs and annual/biennial plants, as well as between perennial forbs and perennial grasses.

At the 25m×25m spatial scale (**Figure 4**), the ungrazed area demonstrated a significant negative association between shrub/semi-shrub groups and perennial grasses, along with a weak negative association between shrub/semi-shrub groups and annual/biennial plants. Weak positive associations were observed between annual/biennial plants and both perennial grasses and perennial forbs. In the heavily grazed area, perennial forbs showed a significant positive association with shrub/semi-shrub groups, while a weak positive association occurred between perennial grasses and annual/biennial plants. All other functional group pairs exhibited negative associations.

At the 2.5m×2.5m spatial scale under ungrazed conditions (**Figure 4**), perennial grasses exhibited positive associations with perennial forbs and shrub/semi-shrub groups but a negative association with annual/biennial plants. Perennial forbs displayed distinct negative associations with shrub/semi-shrub groups and pronounced positive associations with annual/biennial plants. In the heavily grazed area, reduced functional group diversity was observed: perennial forbs showed negative associations with both perennial grasses and annual/biennial plants, while annual/biennial plants maintained a positive association with perennial grasses.

#### 3.4.3 Percentage co-occurrence

**Figure 5:**
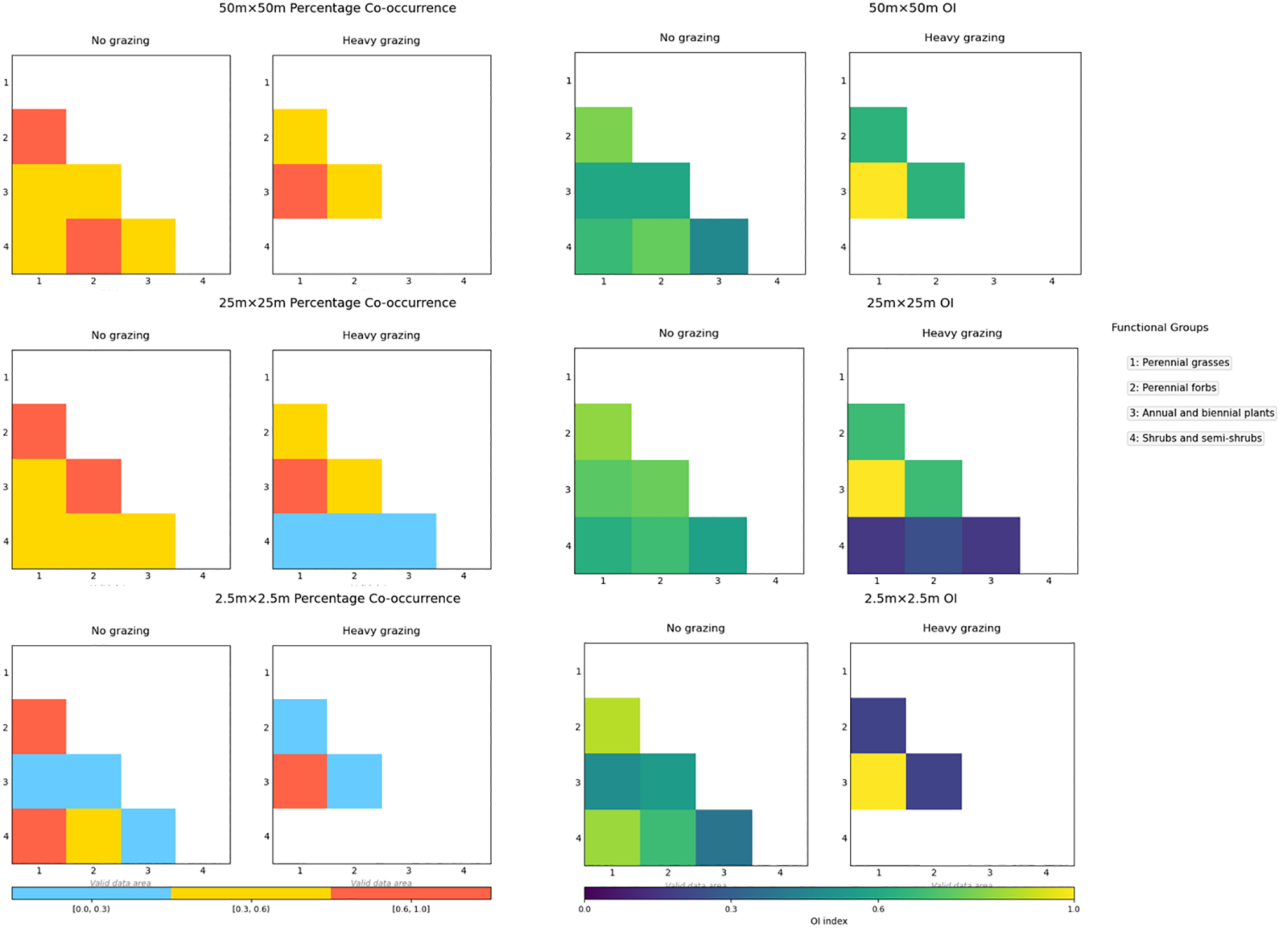
Ochiai index (OI) values and percentage co-occurrence (PC) at different scales under different grazing intensities.

At the 50m×50m spatial scale (**Figure 5**), PC values between perennial forbs and both perennial grasses and shrub/semi-shrub groups in the ungrazed area fell within the range [0.6, 1], while PC values among other functional group pairs ranged between [0.3, 0.6). In the heavily grazed area, PC values between perennial grasses and annual/biennial plants were within [0.6, 1], with all other functional group pairs exhibiting PC values in the [0.3, 0.6) range.

At the 25m×25m spatial scale under ungrazed conditions (**Figure 5**), two functional group pairs—perennial forbs with perennial grasses and perennial forbs with annual/biennial plants—showed PC values within [0.6, 1], whereas PC values for other pairs ranged between [0.3, 0.6). In the heavily grazed area, only the annual/biennial plant-perennial grass pair exhibited PC values in [0.6, 1]. PC values between shrub/semi-shrub groups and both perennial grasses and annual/biennial plants fell below 0.3 ([0, 0.3)), with remaining functional group pairs ranging between [0.3, 0.6).

At the 2.5m×2.5m spatial scale in the ungrazed area (**Figure 5**), PC values for perennial grass-perennial forb and perennial grass-shrub/semi-shrub pairs were within [0.6, 1], while annual/biennial plants displayed PC values below 0.3 ([0, 0.3)) when paired with other functional groups. In the heavily grazed area, reduced functional group diversity was observed: only the perennial grass-annual/biennial plant pair maintained PC values in [0.6, 1], with all other pairs exhibiting PC values below 0.3 ([0, 0.3)).

#### 3.4.4 Ochiai index and dice index

At the 50m×50m spatial scale (**Figure 5**), OI values in the ungrazed area were distributed across the intervals [0.6,1] and [0.3,0.6), whereas in the heavily grazed area, values clustered within [0.6,1], though with reduced functional group diversity compared to ungrazed conditions.

At the 25m×25m spatial scale under ungrazed conditions (**Figure 5**), only the shrub/semi-shrub–annual/biennial plant pair exhibited OI values within [0.3,0.6), while all other functional group pairs showed tightly clustered OI values in [0.6,1]. In contrast, heavily grazed conditions resulted in OI values between annual/biennial plants and other functional groups uniformly declining to the [0.3,0.6) range, indicating weakened associations.

At the 2.5m×2.5m spatial scale (**Figure 5**), the heavily grazed area displayed fewer functional groups than the ungrazed area, accompanied by significantly lower OI values. DI values exhibited congruent patterns with OI values across all scales and treatments.

### 3.5 Interspecific correlation (Pearson correlation coefficient, Spearman’s rank correlation coefficient)

**Figure 6:**
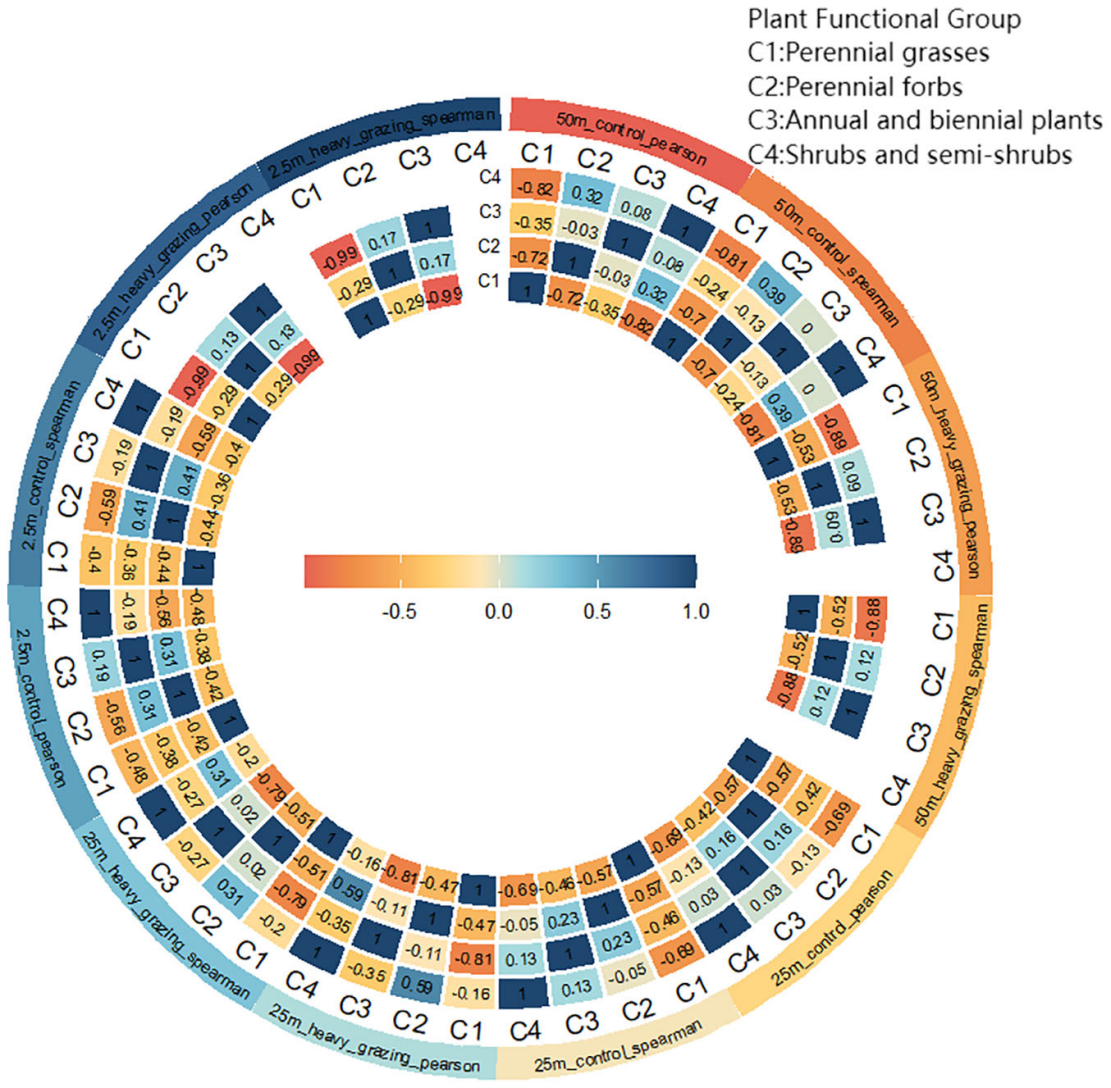
Interspecific correlation (Pearson correlation coefficient, Spearman’s rank correlation coefficient) values at different scales under different grazing intensities.

At the 50m×50m spatial scale (**Figure 6**), Pearson correlation coefficients revealed positive correlations between shrub/semi-shrub groups and perennial forbs, as well as between shrub/semi-shrub groups and annual/biennial plants in the ungrazed area, whereas negative correlations were observed among -all other functional group pairs. In the heavily grazed area, positive correlations occurred between annual/biennial plants and perennial forbs, with negative correlations prevailing among all remaining functional groups.

At the 25m×25m spatial scale (**Figure 6**), Pearson correlation coefficients indicated positive correlations between annual/biennial plants and both perennial forbs and shrub/semi-shrub groups in the ungrazed area, while negative correlations characterized other functional group pairs. In the heavily grazed area, only shrub/semi-shrub groups and perennial forbs exhibited a positive correlation. Spearman’s rank correlation coefficients yielded congruent results with Pearson coefficients across all analyses.

At the 2.5 m × 2.5 m spatial scale (**Figure 6**), Pearson and Spearman correlation coefficients show that only perennial forbs and annual and biennial plants are positively correlated in both ungrazed and heavily grazed areas. In contrast, the remaining functional groups are negatively correlated.

### 3.6 Stability analysis of functional groups

Stability analysis of functional groups using M. Godron’s stability method revealed differential stability patterns across spatial scales and grazing treatments. The importance values show that perennial grasses and perennial forbs are the dominant functional groups, and the number of species of perennial grasses and perennial forbs is high in the experimental area. In contrast, Annual and biennial plants, shrubs, and semi-shrubs show functional group deficiency at smaller spatial scales and under heavy grazing conditions, so their stability is not investigated. Perennial grasses exhibited higher stability in ungrazed areas compared to heavily grazed areas at all spatial scales, though remaining in an unstable state (**Table 5**). Perennial forbs demonstrated greater stability at larger spatial scales (50m×50m), with higher stability in ungrazed areas than in grazed conditions (**Table 6**). The maximum stability for perennial forbs occurred in ungrazed 50m×50m plots, evidenced by an intersection point at (19.97, 80.03), closest to the theoretical stable equilibrium (Davison et al., 2020; Tran et al., 2021). Annual/biennial plants and shrub/semi-shrub groups consistently remained unstable across all spatial scales and grazing treatments. According to the Godron stability method, the functional groups under all treatments did not reach the theoretical stable state (Euclidean distance > 0). However, perennial forbs show a tendency to be closer to the theoretical equilibrium point in no-grazing areas than in heavily grazing areas, and their Euclidean distance (50m×50m scale: 17.9) is significantly smaller than the corresponding value under heavily grazing conditions (50m×50m scale: 37.8). This indicates that although the system as a whole is unstable, the absence of grazing interference enhances the relative stability of this functional group.

**Table 5:** Stability analysis of Perennial grasses at different spatial scales and grazing intensities (M.Godron method).

Spatial scale	Habitat type	Fitting equation	Determination coefficient	Intersection coordinate	Euclidean distance
50m×50m	no grazing	y = -0.0088x^2^ + 1.6610x + 22.4903	R² = 0.9944	(32.66, 67.34)	17.90
	heavy grazing	y = -0.0026x^2^ + 1.2606x + 0.0000	R² = 1.0000	(46.76, 53.24)	37.84
25m×25m	no grazing	y = -0.0041x^2^ + 1.4094x + 0.0000	R² = 1.0000	(44.93, 55.07)	35.26
	heavy grazing	y = -0.0137x^2^ + 2.3671x + 0.0000	R² = 1.0000	(34.54, 65.46)	20.57
2.5m×2.5m	no grazing	y = -0.0138x^2^ + 2.2707x + 9.5426	R² = 0.9863	(31.95, 68.05)	16.90
	heavy grazing	y = -0.0215x^2^ + 3.5945x + 44.2877	R² = 1.0000	(38.26, 61.74)	25.82

**Table 6:** Stability analysis of perennial forbs at different spatial scales and grazing intensities (M.Godron method).

Spatial scale	Habitat type	Fitting equation	Determination coefficient	Intersection coordinate	Euclidean distance
50m×50m	no grazing	y = -0.0011x^2^ + 0.3851x + 72.7907	R² = 0.9980	(19.97, 80.03)	0.05
25m×25m	no grazing	y = -0.0020x^2^ + 0.4855x + 71.8627	R² = 1.0000	(19.46, 80.54)	0.76
	heavy grazing	y = -0.0176x^2^ + 2.7647x + 0.0000	R² = 1.0000	(31.09, 68.91)	15.69
2.5m×2.5m	no grazing	y = -0.0041x^2^ + 0.7797x + 62.2881	R² = 0.9861	(61.80, 38.20)	3.30

### 3.7 Semi-variogram

**Figure 7:**
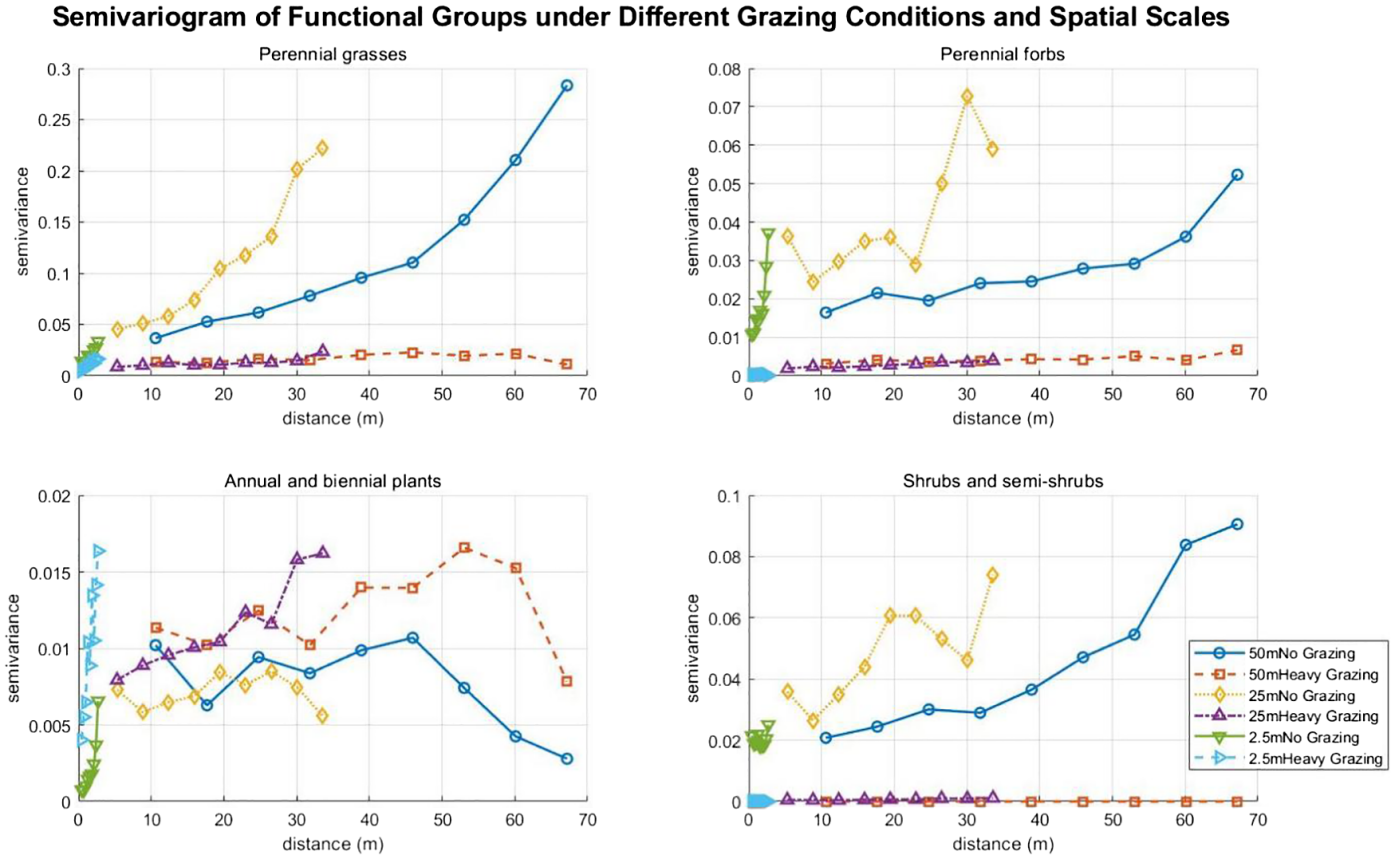
Semivariogram of functional groups under different grazing conditions and spatial scales.

In the absence of grazing, the semivariance of perennial grasses increases significantly with distance, indicating increasing spatial heterogeneity. Under heavy grazing, the semivariance remains low and flat, suggesting that grazing pressure destroys patch structure, leading to either uniform distribution or fragmentation, and suppressing spatial heterogeneity (Bisigato et al., 2005). For perennial forbs, in ungrazed areas of 25m, the semivariance reaches a peak at medium to short distances before declining, implying the presence of local patches at small scales; in 50m ungrazed areas, the semivariance increases slowly, reflecting weak spatial autocorrelation at large scales; under heavy grazing, the extremely low semivariance indicates that grazing pressure eliminates local heterogeneity, resulting in a random distribution (Bisigato et al., 2005) (**Figure 7**). For annual and biennial plants, under heavy grazing, the semivariance increases significantly at medium distances, reflecting how grazing pressure creates microhabitats that promote the establishment of annual plants and the formation of medium-scale patches. The fluctuating and disorderly curves in ungrazed areas indicate weak spatial autocorrelation (**Figure 7**). For shrubs and semi-shrubs, in 50m ungrazed areas, the semivariance continues to increase with distance, demonstrating strong spatial autocorrelation at large scales; in 25m ungrazed areas, the semivariance increases at short distances before declining, suggesting more concentrated shrub patches at small scales; under heavy grazing, the extremely low semivariance shows that grazing pressure suppresses shrub growth and destroys their spatial heterogeneity (Komac et al., 2011) (**Figure 7**).

## 4 Discussion

### 4.1 Analysis of functional group diversity across spatial scales and grazing intensities

Grazing intensity significantly alters plant functional group diversity in *Stipa breviflora* desert steppe, exhibiting pronounced spatial scale dependency. Overall, heavy grazing markedly reduces diversity indices (e.g., Shannon-Wiener, Simpson, Pielou) due to decreased species evenness and simplified functional group structure (Tran et al., 2021). This simplification manifests as overconsumption of dominant species (e.g., *Stipa breviflora*) causing community homogenization, while grazing-tolerant species (e.g., *a*nnual weeds) rapidly colonize released niches to establish dominance (Sachura et al., 2024). Consequently, high-nutritional-value grass functional groups decline, whereas disturbance-resistant, high-fiber plants increase. Diversity responses diverge across spatial scales: At finer scales (2.5m×2.5m), diversity indices are generally lower, constrained by limited environmental heterogeneity and sampling bias—*Stipa breviflora* root competition inhibits coexistence of sensitive species, and sparsely distributed forbs may be undersampled (Ravenek, 2015). Conversely, at intermediate scales (25m×25m), the Margalef richness index shows a distinct increase, likely driven by localized outbreaks of grazing-tolerant species (e.g., *Chenopodium aristatum*) and patch dynamics. Heavy grazing suppresses dominant species expansion, enabling annual plants to rapidly colonize vacated niches and transiently boost species richness (Bajwa et al., 2021). Grazing-induced vegetation patchiness allows this scale to capture more ephemeral species in residual grazing areas, while control plots exhibit lower species counts due to uniform dominance—aligning with the scale-dependent intermediate disturbance hypothesis, where medium scales show greater sensitivity to short-term disturbance responses than broader scales (50m×50m), where spatial averaging masks local variations (Stone, 1995; Mayor et al., 2015; McConaghy, 2016). At broad scales, non-significant diversity differences between grazing treatments likely stem from compensatory effects among functional groups (e.g., spatial complementarity between deep-rooted shrubs and shallow-rooted grasses buffering individual group impacts) (Bakker, 2018; Gamadaerji et al., 2020; Wang et al., 2022). Collectively, grazing regulates diversity dynamics through multi-scale mechanisms: local competition and morphological adaptations [e.g., cuticle thickening in *Cleistogenes songorica* (Sachura et al., 2024)] dominate species turnover at finer scales, while functional complementarity and resource partitioning sustain system stability at broader scales (Guo et al., 2020; Li et al., 2022a; Montoya et al., 2015).

### 4.2 Analysis of interspecific associations across spatial scales and grazing intensities

Grazing intensity and spatial scale interactively reconfigure interspecific associations among plant functional groups in *Stipa breviflora* desert steppe. Variance Ratio (VR) analysis reveals scale-dependent patterns: at finer scales (2.5m×2.5m), heavy grazing generates significantly positive associations (VR > 1) as it suppresses dominant groups (e.g., perennial grasses), releasing niche space for complementary distributions between grazing-tolerant species (e.g., annual/biennial plants) and residual dominants (Tang et al., 2019; Liu et al., 2022). Conversely, at larger scales (50m×50m), ungrazed areas exhibit non-significant negative VR values indicating inherent resource competition, plants achieve resource division through spatial division of their root systems (root domain territorialization), forming symmetrical competition (resource allocation in proportion to biomass), and reducing intraspecific consumption (de Kroon et al., 2003), while heavy grazing homogenizes resource consumption, driving VR toward neutrality (VR = 1) (Le Bagousse-Pinguet et al., 2012). This supports the Competitive Release Hypothesis where disturbances reduce niche overlap (Segre et al., 2016). The fine-scale positive associations under grazing arise from synergistic environmental filtering, habitat heterogeneity, and interspecific facilitation - reflecting adaptive coordination among stress-tolerant species rather than classical competitive antagonism (Magura et al., 2018; Dai et al., 2020; Jiang et al., 2022).

Functional group pairwise associations show distinct scale-grazing interactions: Ungrazed conditions feature non-significant associations at larger/intermediate scales (50m×50m,25m×25m) due to stable coexistence via resource partitioning (Yang et al., 2018; Pescador et al., 2021; Beck et al., 2022; Homulle et al., 2022; Yang et al., 2015; Sinclair et al., 2020), but significant positive perennial grass-forb correlations at fine scales (2.5m×2.5m) via microhabitat root complementarity (Lencinas et al., 2007; Nagel et al., 2015; Liu et al., 2023). Heavy grazing fundamentally disrupts these patterns: at large scales, grazing-tolerant species form positive associations (Cole, 2003) while grass-forb competition intensifies (Fu et al., 2019); at fine scales, amplified competition generates negative associations among most groups (Pescador et al., 2021), though transient grass-annual plant cooperation persists through shared resource exploitation (Nagel et al., 2015; Zhang et al., 2021). Shrub-related associations further demonstrate scale effects: negative grass-shrub correlations at large ungrazed scales [resource competition (Gamadaerji et al., 2020)] shift to positive shrub-forb associations under grazing [collaborative stress tolerance (Alvarez et al., 2011; Puigdefabregas et al., 1999; Thorpe et al., 1998; Saldo and Bartolome Filella, 2021)]. Collectively, grazing reshapes association networks by altering resource distribution and competitive hierarchies (Saiz and Alados, 2012; Zhang et al., 2020; Newman, 2006; Olesen et al., 2007).

Co-occurrence (PC) and association strength (OI/DI) indices corroborate these dynamics: Ungrazed large scales show high PC (0.6–1) and distributed OI values ([0.3,1]), (Dai et al., 2019; Shan et al., 2018) indicating multi-tiered networks sustained by environmental heterogeneity (Zainelabdeen et al., 2021; Rodríguez et al., 2023; Zheng et al., 2024; Zainelabdeen et al., 2020; Ren et al., 2018). Heavy grazing universally reduces PC values and concentrates OI in higher ranges ([0.6,1]) but with fewer functional groups, forming simplified high-co-occurrence/low-biodiversity communities (Saiz and Alados, 2012; Zainelabdeen et al., 2021; Hao et al., 2022; Zainelabdeen et al., 2020; Ren et al., 2018). Scale reduction intensifies these trends at fine scales (2.5m×2.5m), ungrazed communities maintain elevated OI through microhabitat symbiosis (Gamadaerji et al., 2020; Hu et al., 2022; Hu et al., 2020), while grazing collapses associations to near-zero PC/OI values for most pairs, leaving only rudimentary grass-annual plant networks (Saiz and Alados, 2012; Jiménez et al., 2014). Grazing consistently weakens interspecific linkages by reducing species occurrence probabilities, with scale diminution exacerbating this effect (Tsutsumi et al., 2002; Saiz and Alados, 2012; Zhang et al., 2013; Liang et al., 2018).

Pearson correlations integrate these patterns: Positive shrub-forb/annual correlations in ungrazed large scales reflect microhabitat amelioration (Archer et al., 2017; Davison et al., 2020), contrasting with grazing-induced positive annual-forb correlations through complementary resource-use (Herlocker et al., 1999; Mbembok et al., 2017). Intermediate scales strengthen mutualistic networks ungrazed (Setiawan et al., 2023) but only retain shrub-forb synergies under grazing (Bascompte et al., 2003; Bascompte and Jordano, 2007). Critically, large-scale associations are primarily environmentally mediated [heterogeneity-driven resource allocation (Wang et al., 2014; Hai et al., 2021)], while fine-scale linkages are governed by direct biotic interactions (Losapio et al., 2018). Grazing drives transitions from multispecies synergies to simplified tolerance-dominated networks via resource redistribution and physiological stress (Abu Bakar et al., 2023), offering actionable insights: restoring degraded grasslands requires optimizing resource heterogeneity at broad scales while enhancing key species interactions at fine scales (Peters et al., 2006; Wang et al., 2018; Zhao et al., 2023).

### 4.3 Stability analysis of functional groups across spatial scales and grazing intensities

Plant functional groups exhibited distinct stability responses to grazing across spatial scales in Stipa breviflora desert steppe. Perennial grasses showed higher stability in ungrazed areas than heavily grazed plots at all spatial scales yet maintained overall instability. Their long life cycles and regenerative capacity supported greater biomass and structural stability without grazing (Zhang et al., 2020), but grazing directly damaged aboveground tissues (reducing photosynthesis and reproduction), while soil compaction and nutrient depletion in heavily grazed areas further impaired recovery (Hamza and Anderson, 2005). Notably, even ungrazed perennial grasses displayed relatively low stability, likely due to natural disturbances (e.g., climatic fluctuations) and interspecific competition (Rayburn, 2011; Siebert et al., 2020). Perennial forbs reached peak stability at broader scales (50m×50m) under ungrazed conditions [intersection point approaching (Davison et al., 2020; Tran et al., 2021)], where increased environmental heterogeneity enabled adaptive niche strategies (e.g., divergent root architectures and nutrient uptake) to buffer environmental fluctuations (Liang et al., 2022; Ning et al., 2022; Meng et al., 2023). Heavy grazing reduced forb stability by damaging biomass and disrupting soil-nutrient cycles (Giuliani et al., 2024; Hao et al., 2024). Conversely, annual/biennial plants and shrubs/semi-shrubs remained unstable across all scales and grazing intensities: short-lived species exhibited high vulnerability to environmental variability due to brief life cycles and seed-dependent regeneration (Zhang et al., 2020), further compromised by grazing consumption of seedlings/seeds; woody plants suffered from slow growth, browsing/trampling sensitivity, patchy distributions, and weak interspecific interactions (El-Mahi, 1990). These differential responses arise from scale-grazing interactions modulating growth traits, ecological strategies, and disturbance resilience—perennial groups show grazing-sensitive stability (especially at broader scales), while short-lived and woody groups persist unstable throughout the grazing gradient.

## 5 Conclusions

Based on a systematic investigation into the response mechanisms of plant functional groups in the *Stipa breviflora* desert steppe under varying spatial scales and grazing intensities, the following key conclusions were drawn:

1. Grazing intensity significantly influenced plant functional group diversity, with effects exhibiting distinct spatial scale dependence. Heavy grazing generally reduced community Shannon-Wiener diversity, Simpson dominance, and Pielou evenness indices. However, at the intermediate 25m×25m scale, the Margalef richness index increased, indicating that this scale is most sensitive to grazing disturbance—likely due to patchy invasion of grazing-tolerant species and the scale-dependent effects of the intermediate disturbance hypothesis.
2. Interspecific associations showed significant scale-dependent characteristics. At larger scales (50m×50m), interactions were primarily driven by resource competition and environmental heterogeneity, while at finer scales (2.5m×2.5m), direct biological interactions (e.g., facilitation or exclusion) played a dominant role. Heavy grazing markedly altered interspecific association networks, leading to simplified, high-co-occurrence symbiotic structures between perennial grasses and annual/biennial plants, while strongly suppressing perennial forbs and shrubs/semi-shrubs.
3. The stability of different functional groups responded differently to grazing and spatial scale. Perennial grasses and forbs exhibited relatively higher stability under grazing exclusion, particularly at larger scales (50m×50m). In contrast, annual/biennial plants and shrubs/semi-shrubs remained unstable across all treatments, showing high dependence on environmental fluctuations and disturbance conditions.
4. This study underscores the importance of multi-scale analysis in understanding and managing grazing ecosystems. The 25m×25m scale was identified as critical for characterizing grazing impacts and should be prioritized as a key spatial unit for future monitoring and restoration practices. Based on these findings, we propose integrated multi-scale restoration strategies: optimizing resource allocation and habitat heterogeneity at broad scales, and enhancing community stability through functional group combinations and mutualistic species assemblies at fine scales.

In conclusion, this research provides a theoretical basis and practical guidance for multi-scale grazing management in the Stipa breviflora desert steppe, highlighting the necessity of designing spatially explicit restoration measures based on underlying mechanisms in dynamic environments under anthropogenic disturbance.

## Data Availability

The raw data supporting the conclusions of this article will be made available by the authors, without undue reservation.
